# Tertiary Lymphoid Structures in Cancer: Drivers of Antitumor Immunity, Immunosuppression, or Bystander Sentinels in Disease?

**DOI:** 10.3389/fimmu.2017.01830

**Published:** 2017-12-19

**Authors:** Emily Jayne Colbeck, Ann Ager, Awen Gallimore, Gareth Wyn Jones

**Affiliations:** ^1^UCL Cancer Institute, University College London, London, United Kingdom; ^2^Division of Infection and Immunity, School of Medicine and Systems Immunity Research Institute, Cardiff University, Cardiff, United Kingdom

**Keywords:** tertiary lymphoid structures, cancer immunotherapy, high endothelial venules, lymphoid neogenesis, tumor microenvironment

## Abstract

Secondary lymphoid organs are integral to initiation and execution of adaptive immune responses. These organs provide a setting for interactions between antigen-specific lymphocytes and antigen-presenting cells recruited from local infected or inflamed tissues. Secondary lymphoid organs develop as a part of a genetically preprogrammed process during embryogenesis. However, organogenesis of secondary lymphoid tissues can also be recapitulated in adulthood during *de novo* lymphoid neogenesis of tertiary lymphoid structures (TLSs). These ectopic lymphoid-like structures form in the inflamed tissues afflicted by various pathological conditions, including cancer, autoimmunity, infection, or allograft rejection. Studies are beginning to shed light on the function of such structures in different disease settings, raising important questions regarding their contribution to progression or resolution of disease. Data show an association between the tumor-associated TLSs and a favorable prognosis in various types of human cancer, attracting the speculation that TLSs support effective local antitumor immune responses. However, definitive evidence for the role for TLSs in fostering immune responses *in vivo* are lacking, with current data remaining largely correlative by nature. In fact, some more recent studies have even demonstrated an immunosuppressive, tumor-promoting role for cancer-associated TLSs. In this review, we will discuss what is known about the development of cancer-associated TLSs and the current understanding of their potential role in the antitumor immune response.

## Introduction

Secondary lymphoid organs (SLOs) primarily serve to initiate adaptive immune responses to exogenous pathogens. For this, SLOs provide a location for interactions between rare antigen-specific naive lymphocytes and antigen-presenting cells draining from local tissues. The importance of SLOs in mediating homeostatic lymphocyte proliferation and rapid recall responses to returning antigens have long been appreciated (1, 2). Also it is now well recognized that SLOs contribute to peripheral immune tolerance to self-antigens and commensals by regulating trafficking of immunosuppressive Foxp3^+^ regulatory T cells (Tregs), major cellular mediators of peripheral tolerance, and facilitating the deletion of autoreactive T cells by SLO-resident extrathymic Aire-expressing cells (3, 4).

Canonical SLOs comprise the lymph nodes (LNs), the white pulp of the spleen, the appendix (in humans), and mucosal-associated lymphoid tissues (MALTs) including intestinal Peyer’s patches (PPs) and the tonsils. SLOs are strategically placed at distinct, predetermined sites throughout the body, together forming a sophisticated network that facilitates continual immune surveillance of interstitial areas, epithelial and mucosal surfaces, and the blood. Individual SLOs have highly organized, specialized architecture that is specifically adapted to promote the immune cell interactions necessary for immune response initiation (2, 5, 6).

The development of canonical SLOs is a genetically preprogrammed process initiated during embryogenesis. Although LNs and PPs develop prenatally, the organogenesis of MALT, such as bronchial-associated lymphoid tissue (BALT) and more plastic lymphoid tissues, including cryptopatches and isolated lymphoid follicles in the intestine, occurs postnatally. Thus, there exists a continuum of lymphoid tissues, from canonical, constitutive SLOs preprogrammed during ontogeny to highly plastic, inducible and transient lymphoid structures that form later in life (2, 7).

SLO organogenesis is recapitulated in the *de novo* development of tertiary lymphoid structures (TLSs) under pathological circumstances (6, 8, 9). TLSs, also termed ectopic lymphoid-like structures or tertiary lymphoid organs, form at the site of infection or chronic inflammation and have been noted in autoimmune disease, allograft rejection, and more recently cancer (2, 6, 10). Crucially, the clinical significance of TLSs is thought to vary from deleterious to protective, emphasizing the need to better understand the formation and function of these structures, which may be contextually different, before clinical targeting.

In this review, we will compare and contrast TLS neogenesis with the development of a prototypic SLO, the LN. Importantly, we will discuss current knowledge surrounding the function of TLSs, specifically within cancer, and consider the implications for the use of next-generation therapeutics.

## Composition and Organization of a TLS Compared to a Prototypic SLO: The LN

**Figure 1:**
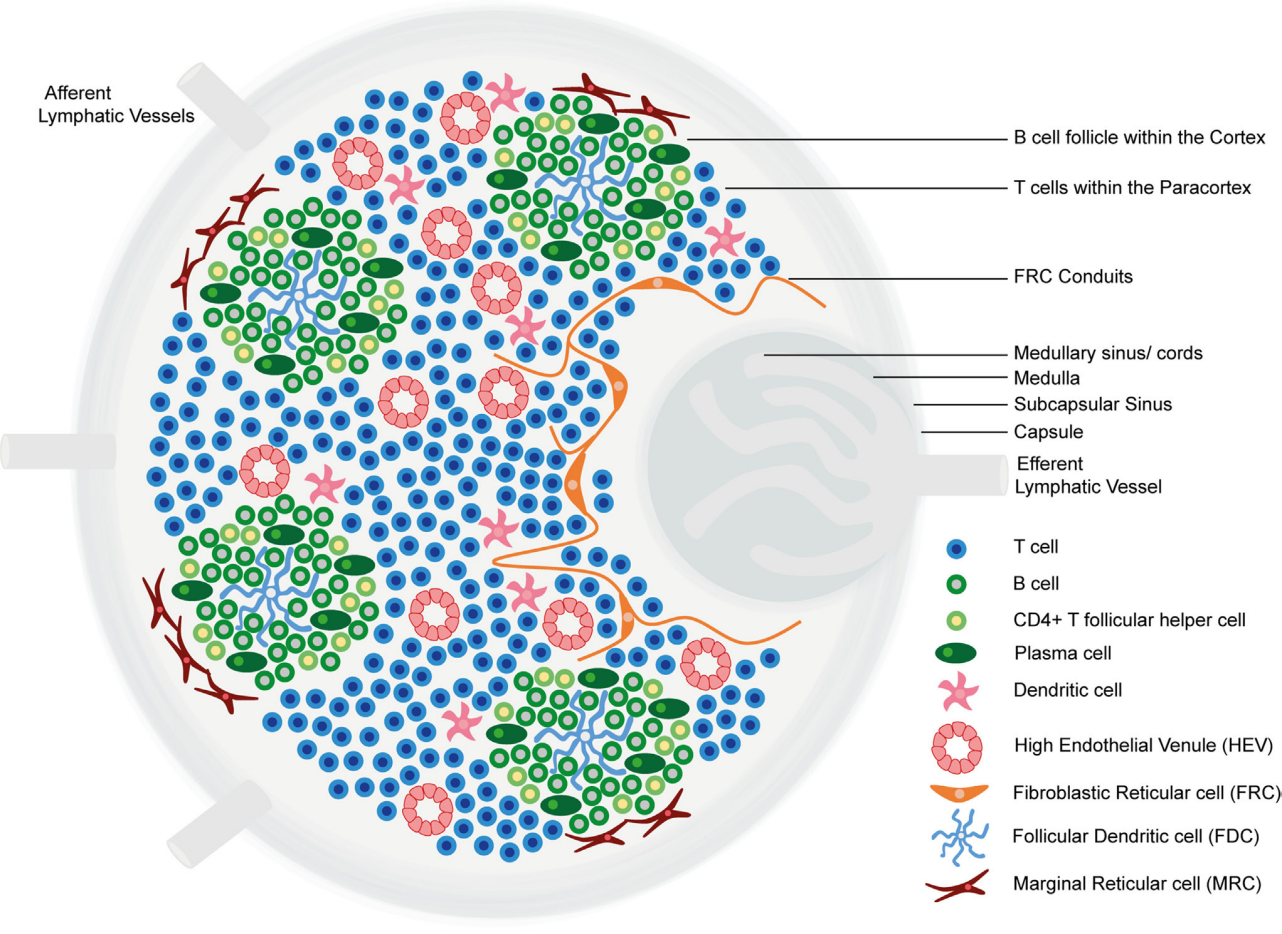
The structure of the lymph node. Lymph nodes comprise a collagen-rich fibrous capsule and an underlying subcapsular sinus (SCS). Cells are segregated into (1) the cortex, consisting of B cells, T follicular helper cells, and follicular dendritic cells (FDCs) arranged in primary follicles, in which B cells survey antigens presented on the FDC stromal network; and (2) the paracortex, which accommodates T cells, dendritic cells (DCs), and fibroblastic reticular cells (FRCs) that form stromal cell networks and reticular fibers, along which T cells and DCs migrate. Upon antigen exposure and stimulation, B cell proliferation within the primary follicle gives rise to germinal centers, containing antibody-producing plasma cells. The inner medulla is composed of lymphatic tissues (medullary cords) separated by medullary sinuses consisting of lymph. FRCs express CCL19 and CCL21, whereas CXCL13 is expressed by FDCs. Marginal reticular cells (MRCs) form a third stromal cell network, situated just under the SCS. Lymph nodes contain lymphatic vasculature and high endothelial venules (HEVs). Afferent lymphatic vessels deliver lymph containing antigen and immune cells, and HEVs are specialized postcapillary venules that primarily deliver naive and central memory lymphocytes.

Lymph nodes comprise an organized collection of immune and stromal cells encapsulated by a fibrous capsule and an underlying subcapsular sinus (SCS; Figure 1) (6, 11, 12). Cells are topologically segregated into a cortex of densely packed B cells and follicular dendritic cells (FDCs) arranged into discrete primary follicles; the paracortex that accommodates less densely packed T cells, dendritic cells (DCs), and fibroblastic reticular cells (FRCs); and the medulla, composed of lymphatic medullary cords, separated by lymph-filled cavities called medullary sinuses. After antigen exposure, B cells proliferate extensively, giving rise to secondary follicles [germinal centers (GCs)] (6). Alongside FDCs and FRCs, marginal reticular cells (MRCs) constitute a third stromal cell network of the LN, situated just under the SCS (13, 14).

LNs contain two vasculature systems: lymphatic vasculature and high endothelial venules (HEVs). Afferent lymphatic vessels deliver lymph, containing antigens and immune cells, primarily DCs, to the SCS (11, 15). From the SCS, lymph percolates through cortical and medullary sinuses and leaves the LN *via* the efferent lymphatic vessel, which delivers lymph to the venous blood (6, 11). HEVs are highly specialized postcapillary venules found in the blood vascular bed within the paracortical region of LNs, the main function of which is homeostatic delivery of naive and central memory lymphocytes from the adjacent bloodstream. Endothelial cells lining HEVs have a distinct plump, cuboidal morphology and express highly specific addressin molecules, collectively termed peripheral node addressins (PNAds) (11, 16). Lymphocytes extravasate through HEV walls according to a multistep adhesion cascade, dictated by the expression of adhesion molecules and chemokines on HEV endothelial cell surfaces (11, 17).

The term “TLS” can refer to structures of varying organization, from simple clusters of lymphocytes, to sophisticated, segregated structures highly reminiscent of SLOs (10, 18–22). TLSs form at localized sites of microbial infection or chronic inflammation and have been noted in many autoimmune diseases and more recently cancer (10). *De novo* development of TLSs is referred to as ectopic lymphoid neogenesis or lymphoid neoorganogenesis (9, 10).

A notable difference between LNs and TLSs is the fact that the former are encapsulated, while the latter represent a congregation of immune and stromal cells confined within an organ or tissue. Crucially, while SLO development represents a genetically preprogrammed process occurring during ontogeny, TLSs form in response to chronic inflammatory cues. Furthermore, while SLOs form at predetermined specified anatomical locations, TLSs typically form in non-lymphoid organs and exhibit plasticity, as they can present transiently, becoming resolved after the elimination of antigen (2, 6). TLS development has been documented in practically all organ settings under conditions of chronic inflammation, including the heart, kidneys, intestine, vasculature system, central nervous system, and bone marrow. The clinical diseases and experimental models in which TLSs have been documented are extensively reviewed in Ref. (10).

Pathologists use specific criteria to define TLSs. According to these criteria, TLSs contain distinct T and B cell compartments, FRC networks and PNAd^+^ HEVs within T cell zones, FDCs and evidence for class switching and reactive GCs in B cell zones, and expression of the enzyme activation-induced cytidine deaminase (AID); an enzyme expressed in GC B cells required for the initiation of somatic hypermutation and immunoglobulin gene class switching (18, 23). Like LNs, TLSs have also been shown to contain lymphatic vessels, although the role of these vessels and whether they resemble afferent or efferent lymphatics of LN vasculature is as yet unknown (4–6). A set of criteria to define *bona fide* TLSs were also recently proposed by Fridman and colleagues (7).

However, many groups apply the term TLS to less well-organized structures (18). Here, we will also use the term “TLS” more loosely. The observed heterogeneity in TLS architecture and organization could reflect disease stage at which biopsies are taken and could therefore represent sampling at varying phases of TLS development, maturation, and/or resolution. Furthermore, we believe that the potential functional resemblance that TLS share with canonical SLOs could be more relevant than precise anatomical structure: these structures have been associated with either deleterious or protective clinical outcomes in human patients, leading to the speculation that TLSs can generate functional adaptive immune responses capable of influencing the progression of disease (10). This will be discussed in detail later.

## TLSs in Cancer

Tertiary lymphoid structures were initially described in the context of non-neoplastic chronic inflammatory conditions, including autoimmune diseases, infections, and idiopathic diseases (6, 18, 21). Neoplastic malignancies share many features with environments of chronic inflammation, including the chronicity of inflammation itself. However, malignant tumors differ from chronic inflammatory environments in one significant aspect that many would assume may preclude the formation of TLSs: the highly immunosuppressive tumor microenvironment (24, 25). Yet, the occurrence of TLSs of varying degrees of organization has been reported in patients afflicted by multiple types of primary and metastatic cancer (Table 1).

**Table 1:** Tertiary lymphoid structures and high endothelial venules in human cancer.

Cancer type	TLS features	Location	Prognostic Value	References
Lung	HEVs	NS	ND	(26)
Lung (non-small-cell)	Compartmentalized T and B cell zones, mature DCs, FDCs, GCs, lymphatic vessels, and HEVs	NS	Favorable (OS, DSS, and DFS)	(27–29)
Colorectal carcinoma	T cells, B cells, and mature DCs	Extratumoral (at invasive margin of tumor stroma)	ND	(30)
	T cells, B cells, and HEVs	Extratumoral (ahead of invasive margin of tumor stroma)	No association (OS)/detrimental (disease stage)	(31)
	Compartmentalized T and B cell zones, GCs, FDCs, HEVs, lymphatic vessels, andlymphoid chemokine expression	Extratumoral (at invasive margin of tumor stroma)	Favorable (DFS, risk of relapse)	(32)
	T cells and mature DCs	NS	Favorable (percent survival and CD3^+^ T cell density within TLS)	(33)
	Compartmentalized T and B cell zones, GCs, FDCs, and DCs	Extratumoral (at invasive margin of tumor stroma) and intratumoral	Favorable (OS and 12-gene TLS signature)	(34)
	Compartmentalized T and B cell zones, mature DCs, and FDCs	Extratumoral (at invasive margin of tumor stroma and adjacent to tumor nests)	ND	(35)
	HEVs	NS	ND	(26)
Colorectal carcinoma lung metastases	T cells, B cells, mature DCs, NK cells, and HEVs	Extratumoral (within tumor stroma)	Favorable (OS and CD8^+^ and mature DC infiltration in TLS)	(36)
Breast carcinoma	T cells and mature DCs	Extratumoral	ND	(37)
	Compartmentalized T and B cell zones, GCs, FDCs, and PCs	Extratumoral (stromal area adjoining tumor nests)	ND	(38)
	Compartmentalized T and B cell zones, FDCs, macrophages, Tfh cells, and GCs	Extratumoral (adjacent to the tumor bed)	Favorable (DFS and 8-gene Tfh signature)	(39)
	T cells, B cells, PCs, and FDCs	NS	ND	(40)
	T cells, B cell, mature DCs, Foxp3^+^ Tregs, and HEVs	Extratumoral (tumor stroma)	Favorable (risk of relapse, MFS, DFS, and OS)	(26, 41)
	T cells, mature DCs, and Foxp3^+^ Tregs	Extratumoral	Detrimental (RFS and OS)	(42)
	Compartmentalized T and B cell zones, GCs, FDCs, Tfh cells, and HEVs	Extratumoral	Detrimental (tumor grade)	(43)
	T cells, B cells, and HEVs	NS	Favorable (pCR)	(44)
Melanoma	T cells, B cells, HEVs, and mature DCs	Extratumoral (at invasive margin of tumor stroma)	Favorable (signs of tumor regression, low Clark level of invasion, and thin Breslow thickness)	(45)
	Activated T cells and mature DCs	Extratumoral (tumor stroma)	Favorable (OS)	(46)
	Compartmentalized T and B cell zones, and CD86^+^ antigen-presenting cells	Intratumoral	Favorable (OS and 12-gene TLS signature)	(47)
	Lymphocytes and HEVs	NS	Favorable (tumor regression and HEV density)	(48)
Prostate cancer	Compartmentalized T and B cell zones, FDCs, CD68^+^ myeloid cells, T-bet^+^ Th1 T cells, Foxp3^+^ Tregs, mature DCs, HEVs, lymphatic vessels, and PCs	Intratumoral	(Phenotypic changes in TLS associated with evanescent prostate carcinomas)	(49)
Cutaneous melanoma metastases	T cells, B cells, mature DCs, FDCs, HEVs, PCs, and GCs	Extratumoral (tumor stroma)	ND	(50)
Ovarian	HEVs	NS	ND	(26)
	CD8^+^ T cells, and antigen experienced atypical memory B cells	Extratumoral (tumor stroma) and intratumoral (tumor epithelium)	Favorable (DSS and CD8^+^/CD20^+^ density)	(51)
	Compartmentalized T and B cell zones, GCs, FDCs, HEVs, DCs, PCs, and Tfh cells	Extratumoral (tumor stroma)	Favorable (DSS and CD8^+^/CD4^+^/CD20^+^/PC density)	(52)
Pancreatic ductal carcinoma	T cells, B cells, mature DCs, and HEVs	Intratumoral and extratumoral	Favorable (intratumoral TLS with OS and DFS)	(53)
Hepatocellular carcinoma	T cells, B cells, neutrophils, NK cells, macrophages, Foxp3^+^ Tregs, FDCs, and HEVs	Extratumoral (non-neoplastic liver parenchyma)	Detrimental (decreased OS/increased risk for late recurrence and histological/12-gene TLS score)	(54)
Testicular seminoma	T cells, B cells, and HEVs	Extratumoral (among tumor epithelial cell nests)	ND	(55)
Primary clear cell renal cell carcinoma	T cells, mature DCs, and HEVs	Extratumoral (at invasive margin)	Favorable (TLS-associated mature DC density with OS and DFS for CD8^high^ patients)	(56)
Diffuse sclerosing variant of papillary thyroid carcinoma	T cells, B cells, GCs, and HEVs	Extratumoral (within tumor stroma)	ND	(57)

*TLS, tertiary lymphoid structure; DC, dendritic cell; Treg, regulatory T cell; FDC, follicular dendritic cell; GC, germinal center; HEV, high endothelial venule; DFS, disease free survival; DSS, disease specific survival; MFS, metastasis free survival; OS, overall survival; pCR, pathologic complete response; RFS, relapse free survival; ND, not determined; NS, not specified*.

Tumor TLSs are largely associated with a favorable clinical prognosis for patients for a number of different solid tumor types. In a retrospective study of 74 early-stage non-small-cell lung cancer (NSCLC) patients, Dieu-Nosjean and colleagues were the first to report the presence of TLSs in patients with lung cancer. The TLSs, referred to as tumor-induced BALT (Ti-BALT), consisted of an organized distribution of DC and T cell clusters and B cell follicles. The researchers demonstrated that the density of mature DC-LAMP (CD208)^+^ DCs, used as a marker of Ti-BALT, correlated with increased overall, disease-specific and disease-free survival (27). The presence of intratumoral HEVs alone is a strong prognostic marker for various types of human cancer, too. Martinet and colleagues demonstrated a significant correlation between the presence of intratumoral HEVs located within lymphocyte-rich clusters and increased disease-free, metastasis-free, and overall survival rates in a retrospective study of 146 invasive breast cancer patients (26). Furthermore, the same group later found an association between tumor-associated HEVs, infiltrating lymphocytes, and tumor regression in malignant melanoma (45). Multiple other studies have provided evidence for an association between TLSs, or HEVs in the absence of TLSs, in tumors and favorable clinical outcome (Table 1).

However, the relationship between tumor-associated TLSs and patient outcome appears to depend on many parameters, including cancer type and disease stage. In our own studies, the majority of colorectal cancer-associated HEVs are found at the tumor invasive margin, where they are associated with lymphoid aggregates containing CD20^+^ B cells and CD3^+^ T cells. However, while extratumoral HEV density correlated significantly with numbers of T cells within the invasive margin, the correlation with T cell densities in the tumor center was weak. In fact, lymphoid aggregates were associated with more advanced (Duke’s C stage) disease and were not associated with a more favorable prognosis; indeed, there was a trend toward higher numbers of lymphoid aggregates in those patients who did not survive >5 years posttumor resection relative to those who did (31). In addition, quantification of TLSs within the non-neoplastic liver parenchyma of 66 patients who had undergone resection for hepatocellular carcinoma (HCC) revealed an increased risk for late tumor recurrence and lower overall survival for patients with a high histological TLS score (54). Furthermore, tumor-associated TLS formation was associated with a higher tumor grade in 290 primary breast carcinoma patients (43). TLSs or isolated PNAd^+^ HEVs have also been documented in diverse mouse models of cancer, including lung adenocarcinoma, HCC, melanoma, and fibrosarcoma (54, 58–61). As is the case for human cancer, the relationship between TLSs/HEVs and tumor control appears to be variable: data evidencing these disparate roles will be discussed in detail later.

## Development of TLSs

**Figure 2:**
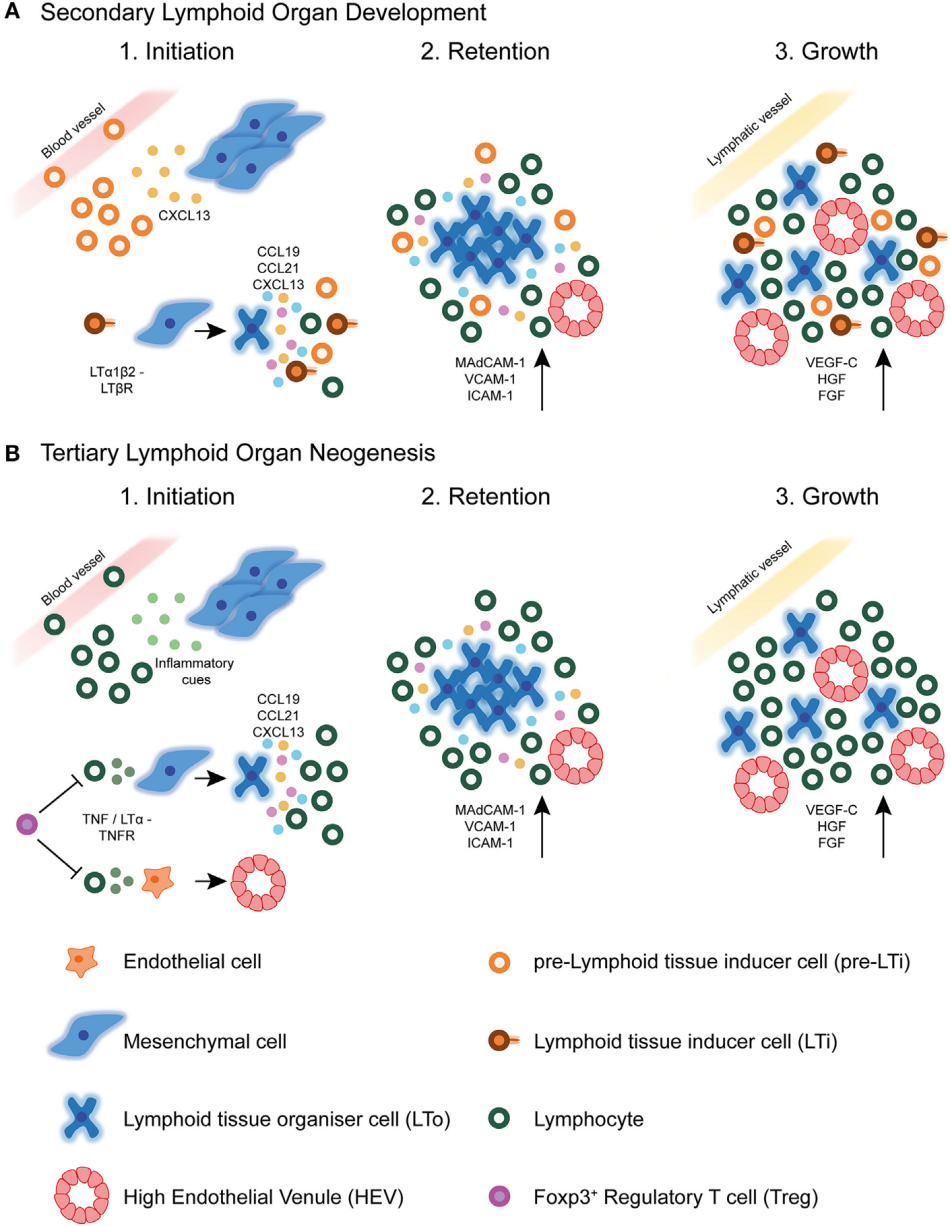
Secondary lymphoid organ and tertiary lymphoid structure development. **(A)** During secondary lymphoid organ development, precursor lymphoid tissue inducer (pre-LTi) cells are initially attracted to the lymph node anlagen from adjacent blood vessels by CXCL13 secreted by resident mesenchymal cells. Clustering of these first cells facilitates crosstalk leading to their maturation into mature LTi cells, which express surface LTα_1_β_2_. Interaction of LTi cells with LTβR expressing stromal cells leads to their differentiation into lymphoid tissue organizer (LTo) cells. Mature LTo cells express chemokines as a result of LTβR triggering, which attract further pre-LTi cells and other hematopoietic cells to the developing lymph node anlagen. Also as a result of LTβR triggering, LTo cells express adhesion molecules, which retain infiltrating hematopoietic cells, eventually leading to lymph node growth. Finally, the developing lymph node fosters formation of high endothelial venules (HEVs), and expression of lymphangiogenic factors aids connection of the lymph node to the surrounding lymphatic vasculature. **(B)** In tertiary lymphoid structure neogenesis in tumors, the initiating cues are likely to be of inflammatory origin and may differ between different tumors. These cues attract circulating lymphocytic cells, such as T lymphocytes and NK cells, which have been shown to initiate HEV development *via* secretion of TNFα or LTα_3_. These cytokines may act directly on TNFR expressing endothelial cells in the tumor microenvironment, causing differentiation of already existing tumor vasculature into specialized HEVs. Whether this event precedes tertiary lymphoid structure neogenesis in tumors is currently unknown. The signals involved in SLO development are also shared with TLS formation, including homeostatic chemokines and adhesion molecules. Little is currently known about the involvement of lymphatic vasculature in TLS development or maintenance. Foxp3^+^ regulatory T cells (Tregs) exert a negatively regulatory role over HEV/TLS development in tumors, potentially *via* direct inhibition of initiating hematopoietic cells including T cells.

Studies of the ontogenic development of SLOs provide a paradigm for understanding TLS formation. Despite structural differences between canonical SLOs and TLSs, we now know that many molecular mechanisms underlying SLOs initiation, development, and maintenance are shared with TLSs formation (10) (Figure 2). For instance, chronically inflamed tissues resident to TLSs are often characterized by the expression of homeostatic chemokines and cytokines reminiscent of SLOs (62). However, a detailed understanding of the exact mechanisms by which TLSs form in various pathogenic circumstances, in particular cancer, is still lacking.

### Control of TLS/HEV Neogenesis by Tregs

A key cellular population recurrently suggested to play a regulatory role in ectopic TLS and/or HEV neogenesis is Foxp3^+^ Tregs. Tregs are highly immunosuppressive T cells that maintain immune homeostasis and promote immunological tolerance to self-antigens. Tregs prevent autoimmunity by keeping in check the activation and expansion of overreactive immune cells, therefore limiting excessive and harmful immune responses (63, 64).

Regulatory T cells are highly enriched in tumors, where they impinge on antitumor immune responses (65, 66). In line with this negative regulatory role, several seminal studies have demonstrated the prevention of tumor development or regression of established tumors, following efficient and selective depletion of Foxp3^+^ Tregs (67–69). In our own studies, Treg ablation in the methylcholanthrene (MCA) carcinogen-induced fibrosarcoma mouse model of carcinogenesis resulted in profound activation of Foxp3^−^ CD4^+^ and CD8^+^ T cells and an overall highly significant reduction in tumor growth rate. However, the response to Treg loss was highly variable, with a large proportion of Treg-depleted animals displaying no significant alteration in tumor control. Critically, successful tumor control was determined by the extent of T cell infiltration into the tumor, which was in turn dictated by the development of ectopic, isolated PNAd^+^ HEVs within the tumor mass. HEVs were only ever observed in a proportion of tumors following Treg depletion, and there was an absolute concordance between HEV presence, high numbers of tumor-infiltrating lymphocytes (TILs), and tumor growth control. In our hands, therefore, depletion of immunosuppressive Tregs is a prerequisite for the development of isolated intratumoral HEVs in MCA-induced fibrosarcomas, implying that Tregs can inhibit neogenesis of HEVs, and possibly TLSs, in tumors (61).

Indeed, highly organized inducible BALT (i-BALT) containing PNAd^+^ HEVs, which normally only develop in response to inflammatory insult in wild-type animals, spontaneously develop in lung tissue of germ-free CCR7^−/−^ mice, which are defective in Treg-mediated immune regulation (70, 71). Adoptive transfer of wild-type but not CCR7^−/−^ Tregs into CCR7- deficient hosts largely interrupted i-BALT formation, and homing of Tregs to peripheral lymphoid organs was essential for their prevention of i-BALT formation (70). A separate study reported the development of spontaneous i-BALT in IL-2-deficient animals, which are devoid of Tregs (72). Furthermore, Foo and colleagues showed that i-BALT that form in response to LPS exposure in the lung was driven by neutrophils and negatively regulated by Tregs (73). Such studies imply a crucial regulatory role for Tregs in the prevention of ectopic lymphoid neogenesis. It will be important in the future to define the mechanisms by which Tregs suppress HEV and/or TLS formation in different scenarios if we wish to develop therapeutic strategies to manipulate these ectopic lymphoid structures.

### Lymphotoxin/Tumor Necrosis Factor (TNF) Signaling in SLO Development and TLS Neogenesis

A body of work has demonstrated that the successful development of LNs critically depends on coordinated interactions between stromal lymphoid tissue organizer (LTo) cells and hematopoietic lymphoid tissue inducer (LTi) cells. These interactions occur *via* signaling between the lymphotoxin (LT) α_1_β_2_ ligand, expressed on the surface of LTi cells, and the LTβ receptor (LTβR), expressed on LTo cells [reviewed in Ref. (2, 74, 75)]. Signaling through the LTβR on gp38 (podoplanin) expressing LTo cells results in the expression of lymphoid chemokines CCL19, CCL21, and CXCL13, which attract migrating hematopoietic cells. Also as a result of LTβR signaling, LN stromal cells express adhesion molecules, including vascular cell adhesion molecule-1 (VCAM-1), intercellular adhesion molecule-1 (ICAM-1), and mucosal vascular addressin cell adhesion molecule-1 (MAdCAM-1), which retain the newly arriving hematopoietic cells, leading to LN growth [reviewed in Ref. (2)]. Furthermore, LTβR signaling induces the expression of vascular growth factors such as the lymphangiogenic factor, VEGF-C, by LTo cells, which aids connection of the developing LN to the surrounding lymphatic vasculature (76). Finally, the developing LN becomes colonized by infiltrating lymphocytes, which are guided to specific zones by homeostatic chemokine expression, giving rise to a highly organized LN [reviewed in Ref. (2)]. Embryonic venous blood vessels play a key role in lymphoid organogenesis by delivering LTi cells, which initiate development, as well as other cellular subsets that mature and maintain HEVs (77). Interestingly, some recently published data have challenged certain aspects of this long accepted scheme and will be discussed in detail later.

Lymphotoxin signaling also appears to play a central role in TLS neogenesis. Overexpression of *Lt*α in the kidney and pancreas using the RIP tissue-specific promoter results in chronic inflammation accompanied by highly organized TLS induction. This study by Nancy Ruddle’s group was one of the first to demonstrate the involvement of the same signaling molecules, namely *Lt*α, that control SLO development in TLS formation (9). The mechanisms by which *Lt*α directs embryonic lymphoid organogenesis and TLS neogenesis in the context of chronic inflammation seem similar, particularly in regard to the induction of chemokine expression (78).

Studies have also strongly implicated LTβ and LTβR in TLS neogenesis. Pancreatic TLSs in RIPLTαβ transgenic animals displayed more distinct T and B cell zone separation, higher chemokine expression, luminal expression of PNAd on HEVs, and increased infiltration of naive L-selectin^+^ lymphocytes, relative to RIPLTα mice (79). Furthermore, the loss of LTα_1_β_2_-LTβR signaling resulted in reversion of many aspects of pancreatic TLS formation, including HEV dedifferentiation, stromal network disruption, and loss of chemokine expression (80). These and multiple other studies have strongly implicated LTα_1_β_2_-LTβR signaling in TLS neogenesis at sites of chronic inflammation, particularly in the development and maintenance of specific TLS structures, namely FDC networks and HEVs (19, 80–86).

Signaling *via* the LTβR has also been implicated in neogenesis of TLSs and/or HEVs in cancer. Targeting of an antibody-LTα fusion protein directly to mouse melanomas resulted in neogenesis of TLS-like lymphoid aggregates including PNAd^+^ HEVs (87). Martinet and colleagues demonstrated that DC-LAMP^+^ DCs were the major producers of LTβ in human breast tumors and that LTβ is overexpressed specifically in tumors displaying a high density of HEVs (41).

The alternative LTβR ligand, LIGHT, is thought to play a redundant role specifically in mesenteric LN development (88). LIGHT has also been implicated in neogenesis of cancer-associated TLSs (89). In breast cancer patients, enhanced expression of LIGHT in ectopically formed TLSs in breast tissue relative to SLOs implies a role for this TNF superfamily member in driving cancer-associated TLS formation (90). Similarly, the expression of LIGHT in a fibrosarcoma cell line resulted in upregulation of CCL21 and MAdCAM-1, facilitating the recruitment of vast numbers of naive CD8^+^ T lymphocytes, which were then sufficiently activated *in situ* to facilitate rejection of established tumors (91). Furthermore, a very recent study demonstrated the ability of LIGHT, if targeted to tumor vasculature by the use of a vascular targeting peptide (VTP), to normalize aberrant blood vessels, induce *de novo* TLS neogenesis, and facilitate influx of endogenous T cells. In combination with checkpoint inhibitor immunotherapy and vaccination, the LIGHT-VTP agent enabled the efficient destruction of tumors previously refractory to immunotherapy (92). These results suggest that, at least in some malignant contexts, LIGHT represents an initiating signal to induce TLS formation in tumors.

Interestingly, studies have demonstrated that TLS neogenesis can be dictated by different signaling circuitries to those classically associated with SLO development. Moyron-Quiroz and colleagues cataloged the presence of primitive TLSs, lacking FDC networks and HEVs, in *Lt*α-deficient mice, which lack LNs and PPs (93–96). More recent studies of tumor-associated TLSs in mouse models of cancer have also suggested a potential departure from canonical SLO development. Peske and colleagues noted the presence of HEV-like vasculature expressing PNAd and CCL21 within lymphocytic aggregates in several murine tumors growing in different anatomical locations in wild-type mice (60). Surprisingly, the authors found that blockade of LTβR signaling in B16-OVA tumor bearing animals had no effect on PNAd expression on tumor vasculature and concurrent trafficking of naive OT-I cells into tumors was even enhanced, implicating no loss of HEV function. Instead, the researchers found that homotrimeric LTα_3_, but not TNF, signaling *via* TNFRs was responsible for the induction of HEV-like vasculature in tumors (60).

Indeed, our own studies of Treg-depleted MCA-induced fibrosarcomas underpin this reliance on TNFR signaling in ectopic HEV neogenesis in tumors. Surprisingly, treatment of animals with an antagonistic LTβR.Fc fusion protein increased intratumoral HEV total area and maintained numbers of infiltrating T cells, suggesting preserved function of the vessels. In striking contrast, administration of a TNFR.Ig fusion protein, anti-LTα monoclonal antibody, or anti-TNFα monoclonal antibody led to a drastic decrease in intratumoral HEV total area, with concomitant effects on T cell infiltration. Therefore, we found that, similar to the case of tumor-associated HEV development in the presence of Treg, TNFR signaling predominates as the governing signaling circuitry modulating HEV development in tumors following Treg depletion (97).

### Chemokine Involvement in SLO Development and TLS Neogenesis

Precursor LTi (pre-LTi) cells that seed the LN anlagen are initially attracted by CXCL13 expressed by local stromal cells (74, 98–100). Mice genetically deficient in CXCL13 or its receptor, CXCR5, display failed LTi clustering and an absence of several peripheral LNs. Lymphatic endothelium-derived CCL21 is able to compensate for the lack of CXCL13 expression in the development of certain LNs by attracting the first LTi cellular clusters (99, 101). However, in general, the CCR7/CCL21 ligand receptor pair seems to contribute only an additive affect to the attractive function of CXCL13, as all LNs form in a normal fashion in mice deficient for the CCR7/CCL21 axis but competent in CXCR5/CXCL13 signaling (102).

Chronically inflamed tissues containing TLSs display significantly increased expression levels of homeostatic chemokines involved in SLO development, including CXCL12, CXCL13, CCL19, and CCL21. All four lymphoid chemokines are individually capable of inducing TLS formation when overexpressed using the RIP in the pancreas (80, 103–106). Conversely, the loss of CXCL13, CXCR5, or CCR7 prohibits the formation of TLSs in disease settings (107–109).

However, the TLSs formed following forced expression of these various chemokines differ significantly in terms of size, cellular composition, and structural organization, revealing defined roles for these chemokines in governing organization and thereby the functionality, of TLSs. For instance, while CXCL13 and CCL21 function in terms of segregation of T and B cells into distinct compartments, CCL19 and CXCL12 primarily seem to facilitate lymphocyte recruitment and positioning of various cell types within the organized TLS. These chemokines seem to function upstream of LTα_1_β_2_ signaling, as shown by induction of LTα_1_β_2_ expression on T or B cells by CCL19, CCL21, or CXCL13 (80, 103). However, the anatomical site of TLS formation and/or specific disease processes may influence the relative involvement of various lymphoid chemokines: while CCL21 overexpression drives TLS formation in the pancreas, ectopic expression of this chemokine in the skin fails to stimulate lymphoid neogenesis (104).

Studies of human cancer patients have additionally implicated chemokines CCL19, CCL21, and CXCL13 in the formation of intratumoral TLSs (24, 28, 110). In human lung carcinoma-associated TLSs, CCL19-expressing cells were found to belong predominantly to the mature DC-LAMP^+^ DC population and are suggested to contribute toward maintenance of the lymphoid structure *via* recruitment of CCR7^+^ immune cells, including naive and central memory T cells, and further, recently activated, DCs. CXCL13 expression in lung carcinoma TLSs was detected specifically on CD21^+^ FDCs within GC-like areas of the TLS. These CXCL13-expressing FDCs were found to colocalize with CXCR5^+^ follicular helper T cell-like cells, suggesting that CXCL13 expression in tumor TLSs could contribute toward generation of humoral immune responses *via* recruitment of CXCR5^+^ immune cells into the GC. As is true for TLSs in inflammatory settings, the CCL21 expression in lung tumor TLSs was found to be restricted to lymphatic vessels. This invites the attractive speculation that CCL21^+^ lymphatic vessels in and around tumor TLSs could provide major trafficking systems guiding activated immune cells to local tumor-draining LNs where systemic protection against metastatic dissemination of primary tumor cells could be established (24, 28). However, there is, as of yet, no formal evidence to support this hypothesis. Overexpression of lymphoid chemokines has also been more recently reported in breast carcinomas, where CXCL13-expressing CD4^+^ T follicular helper cells constitute an important component of breast tumor TLSs. Furthermore, a strong Tfh signature robustly predicted increased patient survival (39).

### Cellular Initiators of SLO Development and TLS Neogenesis

Lymphoid tissue inducer cells were first described in the mouse as a fetal population of hematopoietic cells, essential for secondary lymphoid organogenesis (111–114). LTi cells are CD45^+^ CD4^+^ CD3^−^ c-Kit^+^ interleukin 7 receptor-α (IL-7Rα)^+^ ID2^+^ RORγt^+^ cells that derive from a common progenitor found in the liver (115, 116). The absolute requirement for LTi cells in LN development was demonstrated by the complete lack of LNs in animals genetically deficient in genes required for LTi development and maturation: *Rorc*, which encodes the transcription factor retinoic acid receptor-related orphan receptor-γt (RORγt), and *Id2*, which encodes helix-loop-helix protein inhibitor of DNA binding 2 (ID2) (117–119). The function of LTi cells in the initiation of LN development is absolutely dependent on their expression of LTα_1_β_2_, which is induced by TNF-related activation-induced cytokine receptor (TRANCER) signaling (or IL-7Rα signaling in PP development) (116, 120).

Despite the requirement for LTβR signaling between LTi and LTo cells for LN development, LTi clustering proceeds uninterrupted when LTα_1_β_2_ expression by LTi cells (76, 118, 120, 121) or LTβR expression by LTo cells (122) is prevented. A more recent study demonstrated the ability of TNFα, if expressed above basal levels, to compensate for the lack of LTi cells in *Rorc(*γ*t)*^−/−^ animals in driving organogenesis of several peripheral LNs (those draining the skin), including axillary, cervical, inguinal, and brachial, albeit at lower frequency than in wild-type (123). Therefore, this study contradicts the dogma stating that LTi cells are absolutely required for SLO formation. Although these data imply redundancy in the communication networks governing SLO development, for normal development of LN anlagen in early postnatal life, LTβR signaling is absolutely required suggesting that while the identity of the cell providing the signal may not be as important, the signaling pathway driving secondary lymphoid organogenesis must be preserved (123).

Surprisingly, the loss of LTβR expression in CCL19-expressing mesenchymal LTo cells does not impede LN organogenesis, challenging the existing model delineating a crucial role for LTo cells (124). More recently, the same group led by Burkhard Ludewig have elegantly illustrated that lymphatic endothelial cells (LECs), which form a monolayer of cells lining the SCS in mature LNs, in fact act as the first LTo cells by regulating LTi cell migration and retention; activation of mesenchymal LTo cells only occurred following productive crosstalk between LECs and LTi cells in the LN anlagen (125). These results place LECs above mesenchymal LTo cells in the hierarchical contribution by different stromal cell populations in LN organogenesis. Collectively, such studies challenge the widely accepted dogma of a two-cell type crosstalk scheme involving mesenchymal LTo and hematopoietic LTi cells and illustrate the ongoing gap in our knowledge concerning LN organogenesis.

Experimental data gathered from studies utilizing knockout and transgenic mouse models have indicated cooperation between TNF superfamily members and lymphoid chemokines in the process of lymphoid neogenesis, much as in SLO development (6). However, the identity of both a TNF/LT producing LTi-like cell and a TNF/LT responsive stromal LTo-like cell involved in TLS formation still remains elusive. The recent discovery of an equivalent population of RORγt^+^ LTi cells in the adult, which are members of the innate lymphoid cell (ILC) family, prompted many to speculate that such a population could be directly responsible for TLS formation during lymphoid neogenesis (126, 127). Some studies have even suggested a direct role for adult LTi cells in lymphoid neogenesis. For instance, Meier and colleagues demonstrated that the induction of ectopic lymphoid tissue by overexpression of the gene encoding IL-7 was entirely dependent on the presence of LTi cells, as TLSs failed to develop in the absence of *ROR*γ*t* (128). A separate study, in which adult WT LTi cells were adoptively transferred into neonatal CXCR5^−/−^ mice, demonstrated the ability of these cells to induce *de novo* formation of TLSs in the intestine (127). More recently, natural cytotoxicity receptor (NCR) expressing ILC3 cells were found closely associated with lymphoid aggregates in NSCLC, where they induced LTαβ and adhesion molecule expression, suggesting a potential role in neogenesis of these structures (129).

However, several studies have now provided evidence supporting the notion that TLSs can develop in the absence of canonical RORγt^+^ LTi cells and ILC3 cells. Marinkovic and colleagues used a mouse model in which CCL21 overexpression in the thyroid results in TLS formation, to demonstrate the dispensability of conventional RORγt^+^ cells for ectopic lymphoid neogenesis (106). Deletion of *Id2* resulted in the absence of LNs and PPs as expected, but had no effect on the development of TLSs in the thyroid of CCL21 overexpressing animals. Rather, the authors showed that mature CD3^+^ CD4^+^ T cells were absolutely required for the molecular program that instructs TLS development and suggested that these cells interact with DCs to initiate such a program (106). TLSs also develop in the colon of *Rorc(*γ*t)*^−/−^ animals following inflammatory insult (130). Furthermore, i-BALT forms in the lungs of *Rorc(*γ*t)*^−/−^ animals subjected to pulmonary inflammation (131). Interestingly, *TNF/Rorc(*γ*t)*^−/−^ mice demonstrate development not only of some SLOs but also of TLSs in the absence of LTi cells, provided TNFα signaling is increased. However, although TNFα compensates for LTi cell loss to a certain degree, *Id2* expression as well as LTβR signaling is required for complete secondary lymphoid organogenesis and *de novo* TLS neogenesis (123).

In the study by Rangel-Moreno and colleagues, the investigators concluded that CD4^+^ T cell-derived IL-17 was responsible for i-BALT development by inducing LTα-independent CXCL13 expression (131). Other studies have linked IL-17 and/or T_H_17 cells to TLS neogenesis (132, 133). A recently published study demonstrated the involvement of T_H_17 cells in synovial ectopic lymphoid structure development in both experimental and clinical rheumatoid arthritis (RA). This process was under the inhibitory control of IL-27, a cytokine that is often elevated in the inflamed synovium and serum of certain RA patients (134). Interestingly, the IL-17-producing capability of LTi cells is one of the features that has recently indicated an ancestral relationship between LTi and T_H_17 cells (126, 135). However, the identity of a distinct TLS-inducing cell type remains elusive, with B cells and TNF-producing myeloid cells also representing candidates (123, 130).

Dendritic cells, well known for their role in antigen presentation, are also implicated in the modulation and maintenance of the LN HEV phenotype in the adult, *via* expression of the LTβ ligand. After depletion of CD11c^+^ DCs in adult mice, cellularity and size of peripheral LNs was significantly reduced, expression of HEV markers was downregulated, and homing of lymphocytes to LNs impaired. HEVs reverted to an immature phenotype as a result of a direct interaction between DCs and endothelial cells, presumably *via* LT ligands expressed by DCs and endothelial cell-expressed LTβR (136). Similarly, DC-LAMP^+^ DCs positively correlated with HEV density in breast tumors and were significantly associated with a favorable clinical prognosis (41). The presence of DC-LAMP^+^ DCs was also shown to correlate with HEV density in primary melanoma (45). However, despite clustering of DCs around HEVs in breast cancer, the majority of DCs are located outside basal laminal layers encapsulating HEVs, as in LNs, and are therefore unlikely to be capable of direct contact with endothelial cells; a likely prerequisite for initiation of HEV neogenesis (41).

In the above-mentioned study by Peske and colleagues, there was a distinctive lack of PNAd^+^ tumor vasculature in intraperitoneal B16-OVA tumors of gene-targeted animals specifically devoid of CD8^+^ T cells and RAG2-deficient animals. Furthermore, PNAd-expressing vessels were restored in these tumors to levels observed in WT animals upon adoptive transfer of CD8^+^ T cells to RAG2-deficient mice. In contrast, NK cells could compensate for the loss of CD8^+^ T cells in induction of PNAd^+^ vasculature in subcutaneous B16-OVA tumors, suggesting that these two cellular populations can act in a redundant fashion in particular tumors (60).

Indeed, our own studies of the MCA-induced mouse model of carcinogenesis have shown that conventional CD4^+^ CD3^−^ IL-7R^+^ RORγ^+^ LTi cells are absent from HEV^+^ tumors of Treg-depleted animals (61). Instead, by selectively depleting different subsets of immune cells by monoclonal antibody treatment, we found that CD8^+^ T lymphocytes are the primary cell directing neogenesis of HEVs (97). Hence, rather than a reliance on canonical LTi cells, as is the case for ontogenic SLO development, neogenesis of tumor-associated HEVs, and perhaps thereafter TLSs, appears to rely on cytokine-secreting CD8-expressing or NK effector lymphocytes.

While studies have implied a degree of lymphocyte dependency for the development of a mature HEV phenotype in LNs (137), L-selectin-dependent lymphocyte trafficking to peripheral LNs in RAG-1-deficient mice suggests that the development of functional LN HEVs can proceed in the absence of lymphocytes (138). HEV neogenesis in Treg-depleted MCA tumors and Treg replete B16 tumors could therefore represent a significant mechanistic departure from normal LN HEV development and indeed TLS development in non-malignant scenarios of chronic inflammation (60, 97). We hypothesize that robust ongoing antigenic stimulation provided by tumor-associated antigens (TAAs) leads to sufficient activation of intratumoral lymphocytes, which then secrete the required cytokines to induce HEV differentiation of existing tumor vasculature. In the case of strong antigens, such as OVA, this activation of lymphocytes can override Treg-induced immunosuppression to enable a degree of HEV neogenesis in the presence of these potently immunosuppressive cells. However, depletion of Treg, a type of immunotherapy currently being given to cancer patients in multiple clinical trials, appears to unleash the reins on effector lymphocytes, such that their activation leads to prolific HEV-inducing cytokine production in the tumor bed, robust HEV formation, and concomitant tumor control.

## Function of TLSs in Cancer: Sentinel Bystanders or Active Proprietors of the Immune Response or Immunosuppression?

It has been suggested that, similar to SLOs, TLSs function primarily to potentiate the local immune response at the site of formation. Accordingly, TLSs would have the potential to exacerbate or control disease, depending on the nature of the pathology. By-in-large, the current consensus suggests that there would be rationale for therapeutically potentiating TLS formation in the contexts of microbial infection and malignancy, where exacerbation of local immunity could lead to clearance of infection or rejection of a tumor, but inhibiting the formation of TLSs in chronic inflammation and autoimmunity, where heightened local immune responses would contribute to disease progression (10).

However, TLSs are often documented in pathology by histological examination of tissues, which precludes the gathering of definitive evidence for their functional consequence. The majority of the current data linking TLSs with prognosis in patients or disease progression in animals is correlative by nature. The prognostic association of tumor-associated TLSs or HEVs in the absence of TLSs may be confounded by the fact that their neogenesis often occurs in the context of a robust immune response; the link between these ectopic lymphoid structures and a favorable clinical outcome may be indirect and simply reflect the presence of effector T cells. Conclusive functional data are significantly lacking, without which we cannot be sure as to the precise role of these ectopic lymphoid structures within pathological foci; a prerequisite for effective therapeutic targeting.

Studies have implied the occurrence of an active immune response within TLSs found in autoimmune conditions in both mice and humans, evidenced by AID activity in TLS GCs of Sjorgen’s syndrome patients’ salivary glands, and T cell priming and epitope spreading within TLSs in a mouse model of multiple sclerosis, for example (139, 140). One particular TLS, i-BALT can initiate both humoral and cellular immune responses to protect against influenza infection independently of SLOs in animals lacking spleen, LNs, and PPs (94). These structures were even shown to foster and support immunological memory (93). However, studies addressing the question of tumor-associated TLS function in disease progression have yet to provide definitive conclusive evidence for their role.

Particular TLS features are indicative of the ability to support an immune response to antigen. For instance, HEVs found within human and mouse tumors, either in association with defined TLSs or not, express the same adhesion molecules and chemokines as LN HEVs (PNAd, MAdCAM-1, CCL21, and ICAM-1), presumably endowing them with the ability to interact with and promote the egress of lymphocytes of the naive and central memory phenotype from the bloodstream (28, 55, 57, 60, 61). In a preclinical mouse model of colon carcinogenesis, Di Caro and colleagues were able to demonstrate the migration of GFP-labeled splenocytes to TLSs in the colonic mucosa, suggesting active recruitment of lymphocytes (32). Furthermore, the presence of thin walled vessels expressing typical LN lymphatic markers such as LYVE-1 and podoplanin has been documented in cancer-associated TLSs, indicating a means for the entrance of antigen-presenting cells (28, 32). However, live *in vivo* imaging studies of lymphocytes entering *via* HEVs and antigen-presenting cells entering *via* lymphatic vessels are required to solidify the functional consequence of the presence of such vascular structures.

The presence of tumor-associated TLSs in NSCLC, for which mature DCs serve as a reliable marker, was found to shape the T cell infiltrate toward an activated, Th1 and cytotoxic orientation (29). Interestingly, patients with a high infiltration of CD8^+^ T cells in combination with a high density of TLSs demonstrated significantly improved survival relative to patients with high CD8^+^ T cell infiltration in the absence of TLSs, suggesting TLSs actively license the prognostic value of intratumoral cytotoxic T cells. Similarly, the presence of plasma cells expressing markers of antigen-specific responses within TLSs in ovarian cancer was associated with increased responses of tumor-infiltrating CD8^+^ T cells (52). While such studies of gene expression analysis in humans can only ever offer suggestive evidence for a functional role for tumor-associated TLSs, they do propose that TLSs may educate tumor-infiltrating lymphocytes to control tumors better.

Studies attempting to address the question of whether TLSs are capable of supporting an antigen-specific response to endogenous antigen *in vivo* in animal models have yielded intriguing results. Expression of LIGHT in a fibrosarcoma cell line resulted in upregulation of CCL21 and MAdCAM-1 on tumor vasculature, facilitating recruitment of vast numbers of naive CD8^+^ T lymphocytes, which appeared to then be sufficiently activated *in situ* to facilitate rejection of established tumors (91). Furthermore, TLS aggregates induced in mouse melanoma lesions by targeted LTα expression by tumor cells seem to be able to foster an active antitumor immune response in the absence of all canonical SLOs *in vivo*: Schrama and colleagues documented retarded tumor growth and even tumor regression in splenectomized LTα^−/−^ animals. Furthermore, endogenous CD8^+^ T cells specific for the melanoma-associated antigen TRP-2 were detected by *in situ* tetramer staining only within tumors of LTα^−/−^ animals in which TLSs had been induced (141). While previous studies had demonstrated the ability of B16 melanoma tumors to foster T cell priming in the absence of SLOs in LTα^−/−^ mice (142), the study by Schrama and colleagues was one of the first studies to specifically associate this capability with the presence of intratumoral TLSs.

However, it is possible that tumor-associated TLSs are not essential for *in situ* T cell priming in all cases. The absence of fully formed TLSs in subcutaneous tumors that support HEV neogenesis does not appear to influence the ability of these tumors to facilitate *in situ* priming and activation of tumor-specific naive T lymphocytes (60, 61, 97). It is possible that once lymphocytes have successfully accessed the tumor site, *via* lymphoid-like vasculature, priming and initiation of an *in situ* immune response can occur without the support of an organized lymphoid structure. However, this may require activating signals that are not present in every tumor microenvironment: in the case of LIGHT expressing fibrosarcomas, the dual role of LIGHT as a potent costimulatory molecule for T cells in combination with tumor-associated antigens could suffice for robust T cell priming and expansion (91).

Coronella and colleagues described the presence of sophisticated TLSs within human infiltrating ductal carcinoma of the breast, including segregated T and B cell zones with GCs and interdigitating FDCs (38). What is more, the researchers demonstrated a preponderance of clonal intratumoral B cells, relative to peripheral B cells, by sequencing IgG1 heavy chains isolated from three tumors. Analysis of somatic hypermutation levels and patterns were suggestive of affinity maturation occurring within TLS GCs. These findings were later supported by another independent study of breast carcinoma (40) and a separate study of metastatic melanoma (50). Indeed, GCs in LNs are indicative of an active immune response, and studies have demonstrated a humoral immune response associated with TLS GCs in human lung cancer (143) and a correlation between patient survival and TLS GC makers in breast cancer patients (39). These data indicate that tumor-associated TLSs have the capacity to support *in situ* oligoclonal B cell responses driven by tumor tissue associated antigens.

The cellular composition, organization, and localization of tumor-associated TLSs may dictate whether these structures confer an advantageous or deleterious outcome for disease progression. Following on from the observation that the density of DC-LAMP^+^ mature DCs, as a marker of TLSs, correlated with long-term survival in NSCLC patients (27), Germain and colleagues went on to show that a high tumor follicular B cell density correlates with increased survival and that this prognostic value is enhanced when follicular B cells are present in combination with high mature DC densities (143). However, these analyses were conducted across the entire tumor area and not restricted to tumor-associated TLSs, precluding any inference regarding immune responses specifically ongoing within TLSs. In a separate study, García-Hernández and colleagues found a dramatic change in the cellular composition of prostate cancer-associated TLSs in patients that experienced spontaneous tumor regression: tumor-associated TLSs in patients with evanescent prostate cancer consisted of lower frequencies of Tregs and greater frequencies of T-bet^+^ Th1 T cells than those in patients with more advanced disease (49). This dichotomy in TLS composition between immunostimulatory and immunosuppressive components could be crucial in dictating the immunological outcome of these tumor-associated structures.

In accordance with their well-defined immunosuppressive role, the recruitment of Foxp3^+^ Tregs and myeloid derived suppressor cells (MDSCs) to TLSs within B16 melanomas engineered to express CCL21 led to promotion of tumor growth (59). In addition, high numbers of Foxp3^+^ Tregs within lymphoid aggregates surrounding primary breast tumors was indicative of an increased risk of disease relapse and death (42). Furthermore, Tregs within tumor-associated TLSs actively suppressed antitumor immune responses in a mouse model of lung adenocarcinoma (58). These studies demonstrate that tumor-associated TLSs are sometimes associated with immunosuppression rather than immune activation. It is possible, therefore, that in the absence of a strong stimulus provided by tumor neoantigens or without adoptive transfer of transgenic T cells targeted to known tumor antigens, these ectopic lymphoid structures can foster immunosuppression and support rather than limit tumor growth. Indeed, in a mouse model of chronic hepatitis driven by constitutive IKK-NFkB signaling in hepatocytes, which develop aggressive malignant HCC (IKKβ(EE)^Hep^ mice), researchers noted the development of TLSs highly reminiscent of human hepatic TLSs associated with HCC (54). Importantly, hepatic TLSs were found to foster HCC progenitor cells in the mouse model and depletion of TLSs by ablation of adaptive immunity *via* crossing IKKβ(EE)^Hep^ mice to lymphocyte deficient *Rag1*^−/−^ mice substantially attenuated hepatocarcinogenesis. Hence, in certain cancers, tumor-associated TLSs may even serve as immunological microniches promoting the generation of progenitor cancer cells, rather than an effective antitumor immune response.

Importantly, in the study by Gobert and colleagues, the specific location of Treg infiltration in primary breast tumors proved critical to the prognostic value of this observation; Treg presence within the tumor bed itself did not influence disease evolution. However, Tregs present within tumor-associated TLSs displayed a highly activated phenotype, suggestive of their *in situ* activation in response to TAA presented within the TLSs (42). Collectively, these data are indicative of local suppression of T cell responses by activated Tregs within tumor-associated lymphoid structures rather than within the tumor mass itself, supporting the idea that TLSs are active sites of immune responses.

Crucially, depletion of Tregs within TLSs of mouse lung adenocarcinomas led to enhanced costimulatory capacity of DCs, T cell proliferation, and protective antitumor immune responses leading to tumor regression (58). Not only does this study support the developing hypothesis that intratumoral TLSs can represent sites of active local adaptive immunity against tumor but it also highlights a potential requirement to overcome the profound immunosuppression within the tumor microenvironment to license an effective antitumor response fostered by TLSs. Indeed, in our own studies, HEVs that develop in the absence of Tregs in MCA-induced fibrosarcomas are associated with significantly higher intratumoral T lymphocyte frequencies and reduced tumor growth rates (61, 97). While HEVs have been documented in other mouse models of cancer in the presence of Tregs, amplification protocols are required to visualize HEVs and tumor growth control is negligible despite adoptive transfer of high numbers of transgenic T cells reactive to tumor-expressed antigen (60). It is possible therefore that in the absence of strong antigenic stimulation (which are provided in transgenic T cell models of cancer) or immune activation, HEVs and tumor-associated TLSs could foster immunosuppression over antitumor immunity.

Indeed, the precise location of TLSs in regard to the tumor mass may have important implications for the prognostic value of these structures. Tumor-associated TLSs and/or HEVs can be extratumoral, positioned at or outside the tumor invasive margin, or intratumoral, situated within the true tumor mass or tumor nests. In colorectal carcinoma (CRC), PNAd^+^ HEVs are rarely found within the tumor stroma or epithelium and are instead mainly situated in the surrounding extratumoral area (31). The lack of an association between HEVs and prognosis in CRC could suggest that extratumoral lymphoid neogenesis may be indicative of an immune response driven by tumor and one therefore in support of cancer progression and immune evasion. Other studies documenting HEVs truly embedded within the tumor stroma have found a positive prognostic value associated with these structures (26, 45). These discrepancies could also be reconciled by the importance of TLSs/HEVs in enabling infiltration of T cells into the tumor; extratumoral HEVs were not associated with increased TIL frequencies (31), whereas intratumoral HEVs were (26, 45). Similarly, while extratumoral TLS density was not a prognostic marker in pancreatic cancer patients, intratumoral TLSs functioned as an independent favorable prognosticator (53). Furthermore, in support of the idea that the function and therefore prognostic significance of TLSs/HEVs differs with disease stage also, there is no association between extratumoral HEVs in advanced (Dukes’ C, or stage III) CRC tumors and prognosis while lymphoid aggregates in stage II CRC (with no LN involvement) are associated with a favorable prognosis (31, 32). Hence, tumor-associated TLSs/HEVs may only function in the antitumor response during early disease stages, a capability that may be lost during cancer progression in parallel to the establishment of an immunosuppressive tumor microenvironment and loss of tumor immunogenicity. Finally, the primary tumor origin of distal metastases appears to significantly influence the clinical impact, and hence presumably the immune response, of metastasis associated TLSs: T cell and DC infiltration in TLSs of lung metastases of colorectal carcinomas is a predictor of longer overall survival but appears to correlate with poor survival in lung metastases of renal cell carcinoma (RCC) (36, 56). Collectively, these observations indicate that TLS/HEV location in relation to the tumor mass, disease stage, and tumor origin may all be absolutely crucial in dictating the resulting immune response to tumor.

**Figure 3:**
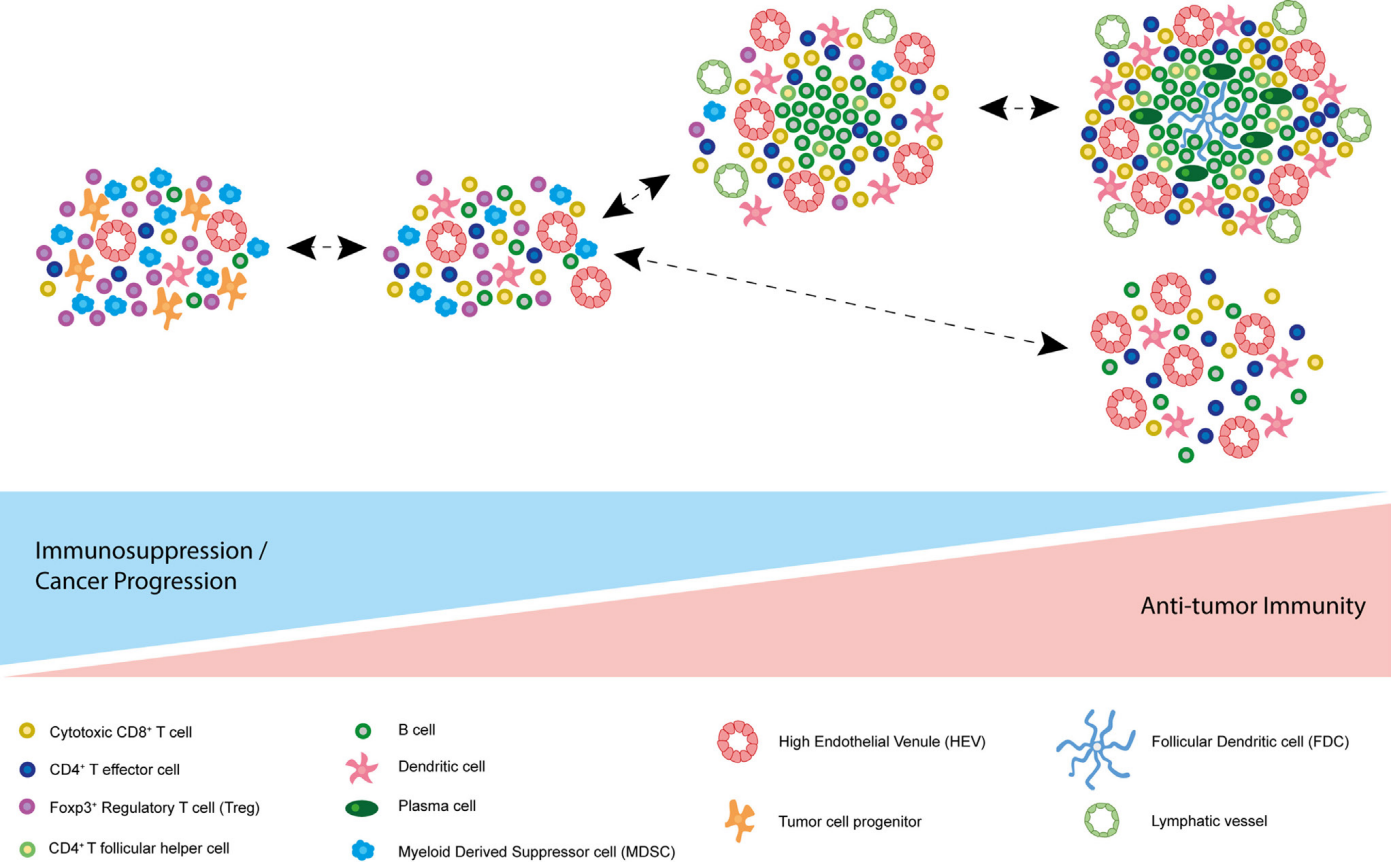
Model of spectrum of tumor-associated tertiary lymphoid structures (TLSs). Based on current literature, it is possible that the function of different tumor-associated TLSs differs due to their composition. At one end of the spectrum, accumulation of immunosuppressive Foxp3^+^ regulatory T cells (Tregs) and myeloid derived suppressor cells (MDSCs) may promote immune tolerance to the tumor. In some cases, TLSs have been described as immunological microniches, which foster malignant progenitor cells, thereby driving cancer progression. At the other end of the spectrum, many cancer-associated TLSs appear to correlate with improved survival and disease regression in multiple human malignancies and animal models. TLS characteristics associated with a good prognosis and an antitumor immune response in addition to cytotoxic CD8^+^ T cells and CD4^+^ effector T cells include plasma cells, T follicular helper cells, B cells, follicular dendritic cells (FDCs), mature DC-LAMP^+^ dendritic cells, and PNAd^+^ high endothelial venules (HEVs). Of note, these differing states of tertiary lymphoid neogensis are not mutually exclusive and can co-exist in the same tumor at the same point in time.

These data underpin the importance of assessing the functional consequences of TLSs and/or HEVs in the absence of TLSs in different cancers prior to therapeutic intervention. It appears that tumor-associated lymphoid structures are capable of supporting an effective immune response in certain contexts. However, a word of caution is issued by studies demonstrating an immune evasive and even disease-promoting role for TLSs (Figure 3). It could be that robust immune system activation, for instance *via* Treg depletion, is required for the beneficial role of intratumoral lymphoid aggregates and vasculature to be unmasked, and encouraging ectopic lymphoid-like vasculature in combination with such therapies may induce an effective immune response to eradicate tumor.

## Concluding Remarks: Toward Therapeutic Targeting of TLS or HEV Neogenesis in Cancer

Observed correlations between cancer-associated TLSs or HEVs in the absence of TLSs and favorable prognosis in several human malignancies have provided the rationale for therapeutically targeting TLSs in an attempt to drive an effective antitumor immune response. Agents directed against key signaling molecules now known to be involved in TLS development have already entered the clinical arena. For instance, clinical trials are investigating the efficacy of intratumoral injections of autologous DCs transduced to express CCL21 in stage IIIB and IV and recurrent NSCLC patients (NCT00601094 and NCT01574222). Although these trials’ primary aim is to determine safety, best dose, and side effects of treatment, secondary objectives are to monitor changes in the infiltrating immune cell populations by immunohistochemistry, which may provide insights into TLS neogenesis within tumors before and after treatment, and whether this will in turn correlate with improved antitumor immunity.

However, as we have learnt, many of the signaling circuitries are overlapping with those essential for SLO development and maintenance, making selective targeting challenging. One signaling pathway that our group and others have identified as playing a major role in HEV neogenesis in tumors but little role in canonical SLO formation is the TNFR signaling pathway (60, 95, 97, 144, 145). There is evidence to suggest that anti-TNF therapy can reverse aspects of TLS neogenesis in RA patients (146), which could account, at least in part, for the therapeutic efficacy of this treatment. Therefore, this pathway represents a potentially selective targetable axis *via* which HEV neogenesis, and possibly TLS development, could be encouraged in tumors. Targeting of TNFα to the tumor vasculature has already been shown to upregulate adhesion molecule expression on the surface of endothelial cells and subsequently enhance CD8^+^ cytotoxic T cell infiltration (147). It will be intriguing to determine whether targeting of TNFα to the tumor, or otherwise stimulating TNFR signaling at this site, may encourage differentiation of tumor vessels toward an HEV-like phenotype, and whether this will in turn enhance T cell infiltration further, and/or antitumor responses.

This review aims to tell a cautionary tale, however: it is becoming clear that lymphoid neogenesis is a highly complex process, which may have wide ranging implications, from antitumor immunity-promoting to immunosuppression. Recent studies have even highlighted an active disease-promoting role for intratumoral TLSs (54). It appears that the function of tumor-associated lymphoid structures may be dictated by their cellular composition and the surrounding immune contexture. It is obvious that we must learn more about the function of these ectopic structures in different human malignancies before we attempt to induce their formation in people. It could be that lymphoid structures within tumors may only serve to promote the antitumor immune response in the context of profound immune activation, a state that can be induced by depletion of immunosuppressive cells such as Tregs and MDSCs. Treg-depleting therapies such as low-dose cyclophosphamide, PI3Kδ inhibitors, and IDO inhibitors are already being used in the clinic for the treatment of human cancers or progressing through clinical trials (148–152) (NCT00567931 and NCT01042535). It is of utmost importance for us to establish whether these treatments alone can induce HEVs or TLSs in tumors. It could be that combining immune-modulating agents with HEV/TLS-targeted therapies may represent the recipe for ultimate effective immune-mediated tumor control.

## Author Contributions

All authors contributed to the conceptualization and design of the work. EJC wrote the article and designed the figures. All authors made significant contributions to the editing and reviewing of the manuscript at multiple stages.

## Conflict of Interest Statement

The authors declare that the research was conducted in the absence of any commercial or financial relationships that could be construed as a potential conflict of interest.

## Abbreviations

SLO, secondary lymphoid organ; TLS, tertiary lymphoid structure; MALT, mucosal-associated lymphoid tissue; LN, lymph node; PP, Peyer’s patch; NALT, nasal-associated lymphoid tissue; BALT, bronchial-associated lymphoid tissue; CCL, CC motif chemokine ligand; CCR, CC-chemokine receptor; CXCL, CXC motif chemokine ligand; CXCR, CXC-chemokine receptor; FDC, follicular dendritic cell; HEV, high endothelial venule.
